# Quantification of Polyphenols in Seaweeds: A Case Study of *Ulva intestinalis*

**DOI:** 10.3390/antiox8120612

**Published:** 2019-12-03

**Authors:** Marie Emilie Wekre, Karoline Kåsin, Jarl Underhaug, Bjarte Holmelid, Monica Jordheim

**Affiliations:** 1 Department of Chemistry, University of Bergen, Allégt. 41, N-5007 Bergen, Norway; 2 Alginor ASA, Haraldsgata 162, N-5525 Haugesund, Norway; 3 Faculty of Chemistry, Biotechnology and Food Science, Norwegian University of Life Science, Universitetstunet 3, N-1433 Ås, Norway

**Keywords:** seaweeds, green algae, marine algae, *Ulva intestinalis*, *Enteromorpha intestinalis*, quantification, polyphenols, flavonoids, apigenin, accelerated solvent extraction, ASE, HPLC-LRMS, HPLC-HRMS, HPLC, TPC, Folin–Ciocalteu, TFC, qNMR

## Abstract

In this case study, we explored quantitative ^1^H NMR (qNMR), HPLC-DAD, and the Folin-Ciocalteu assay (TPC) as methods of quantifying the total phenolic content of a green macroalga, *Ulva intestinalis*, after optimized accelerated solvent extraction. Tentative qualitative data was also acquired after multiple steps of purification. The observed polyphenolic profile was complex with low individual concentrations. The qNMR method yielded 5.5% (DW) polyphenols in the crude extract, whereas HPLC-DAD and TPC assay yielded 1.1% (DW) and 0.4% (DW) respectively, using gallic acid as the reference in all methods. Based on the LC-MS observations of extracts and fractions, an average molar mass of 330 g/mol and an average of 4 aromatic hydrogens in each spin system was chosen for optimized qNMR calculations. Compared to the parallel numbers using gallic acid as the standard (170 g/mol, 2 aromatic H), the optimized parameters resulted in a similar qNMR result (5.3%, DW). The different results for the different methods highlight the difficulties with total polyphenolic quantification. All of the methods contain assumptions and uncertainties, and for complex samples with lower concentrations, this will be of special importance. Thus, further optimization of the extraction, identification, and quantification of polyphenols in marine algae must be researched.

## 1. Introduction

Marine macroalgae, or seaweed, is a large group of macroscopic organisms that are an important component in aquatic ecosystems. The wide diversity of marine organisms is being recognized as a rich source of functional materials and, in 2015, the global seaweed aquaculture production reached 30 million tons [1]. Although marine algae have gained increasing attention over the last years due to the fact of their bioactive natural substances with potential health benefits, they are still identified as an underexploited resource [2,3,4,5,6].

Natural antioxidants with multifunctional potential are of high interest, and numerous studies have focused on natural antioxidants, including polyphenols and flavonoids, from terrestrial plants [7,8,9]. However, the application potential of polyphenolic analyses of marine sources suffers from several factors, most importantly, the lack of exactness with respect to quantitative and qualitative data at a molecular level. Marine plant material with analytic matrices at very low concentrations and a high and variable dissolved salt concentration makes polyphenol analyses challenging [4,10]. The diversity of phenolic compounds also varies from simple to highly polymerized substances which makes qualitative and quantitative procedures, involving sample preparation and extraction, difficult to standardize. Thus, this makes for a further challenge in the analyses and in furthering the research in this field.

Colorimetric assays, such as Folin-Ciocalteu, have been extensively used to quantify phlorotannins and polyphenolic content in seaweeds. However, since the assay is difficult to standardize and not selective, it has been recommended to use the assay for approximate measurements of an extract’s antioxidant potential only [11,12,13,14,15]. Since the colorimetric assays neither separate nor give a correct quantitative measurement of the individual compounds, high-performance liquid chromatography (HPLC) has been the method of choice for separation and quantification of polyphenols in plants. The HPLC with multiple diode array UV-Visible detection (DAD) quantifies according to Lambert-Beer’s law (*A* = *εcl*). A compound’s ability to absorb UV-Visible light (*A*) is related to the compound’s molar absorptivity value (ε) and molar concentration (*c*). The diversity of molar absorptivity values of polyphenols is almost as large as the number of polyphenols existing; even within the same polyphenol class, there will be differences [16]. In the lack of commercially available standards, one standard is often chosen when total amounts of polyphenols or phlorotannins are quantified. Gallic acid (GA) seems to be the most used standard for total polyphenolic quantification and phloroglucinol (PG) for the phlorotannin quantification in brow algae [17,18,19,20]. In addition to the limitations with commercially available standards, HPLC will also suffer from a lack of separation of complex extract matrices and loss of compound amounts due to the irreversible retention on the HPLC column during elution.

In recent years, quantitative ^1^H NMR (qNMR) have gained increasing attention as a method for quantitative determination of metabolites in complex biological matrices [21,22,23]. According to the review by Pauli et al. (2012) [22] and references therein, qNMR methods have proven successful when standard chromatographic methods have been ineffective [22]. In general, qNMR can be considered a primary ratio method of measurement in which the analytes can be correlated directly to a calibration standard, and since the reference compound differs from the analytes, generating a calibration curve becomes unnecessary. However, the quantification needs to be validated with reference compounds. Some work on quantification of phlorotannins in brown algae (*Ascophyllum nodosum*, *Fucus vesiculosus*, and *Cystoseira tamariscifolia*) with qNMR has been done using internal standards [14,23].

In this case study, we examined the polyphenolic content of the green algae *Ulva intestinalis* (syn. *Enteromorpha intestinalis*) collected on the west coast of Norway. An optimized extraction of the polyphenolic content was performed. The extract and semi-purified fractions were further analysed utilizing qNMR with an external reference for quantification of the total phenolic content. For comparison, HPLC-DAD and TPC assay analyses were also performed. To further explore the diverse group of polyphenols in *Ulva intestinalis*, qualitative analyses were performed with HPLC-DAD, HPLC-LR, and HR-MS. We entered this case study with the overarching goal of examining which analytical methods could lead to a more reliable value of polyphenolic content in seaweed and, thus, obtain a better view of the grand potential of seaweed phenolics.

## 2. Materials and Methods

### 2.1. Plant Materials 

Samples of *Ulva intestinalis* (syn. *Enteromorpha intestinalis*) were collected in June from the western coast of Norway; Rogn, Ormhilleren (60°29’38.8” N 4°55’11.9” E). The voucher specimen of *Ulva intestinalis* was deposited in the Herbarium BG (Voucher no. BG-A-75) at the University Museum of Bergen, Bergen.

### 2.2. Chemicals

All chemicals used were of analytical grade. Methanol (≥99.9%), acetonitrile (≥99.8%), trifluoroacetic acid (TFA) and Folin-Ciocalteu reagent were all acquired form Sigma-Aldrich (Sigma-Aldrich, St. Louis, MO, USA). Formic (98–100%) and acetic (99.8%) acids were both acquired from Riedel-de Haën (Honeywell Inc., Charlotte, NC, USA). Luteolin, apigenin, myrcetin, diosmetin, quercetin, caffeic acid, coumaric acid, ferulic acid, sinapic acid, and gallic acid reference standards were all purchased from Sigma–Aldrich (Sigma-Aldrich, St. Louis, MO, USA). The analytical standard of tricin was purchased from PhytoLab (PhytoLab BmbH & Co. KG, Vestenbergsgreuth, Germany), (+)-catechin was purchased from USP (USP, Rockville, MD, USA), and DPPH free radical was purchased from Merck (Merck, Kenilworth, NJ, USA). Deionized water was deionized at the University of Bergen (Bergen, Norway).

### 2.3. Extraction and Purification 

The collected plant material was washed thoroughly in fresh water and air dried. Dried plant material was stored at −20 °C when not used. Dried material was extracted using ASE (Accelerated Solvent Extraction) (Dionex™ ASE™ 350, Thermo Fisher Scientific, Waltham, MA, USA). A dried sample of *Ulva intestinalis* (55.9 g) was mixed with Dionex ASE prep DE sand and added to 66 mL stainless-steel cells with two glass fiber filters placed at the bottom end of the cell, before being extracted using a Dionex ASE 350 Accelerated Solvent Extractor. The extraction procedure consisted of two different methods, one being a pre-soak method, and the other being the primary extraction method. Pre-soaking consisted of extraction at 23 °C under 1500 psi. The static extraction period was 1 min with a flush volume of 50% of cell volume, purged with N_2_ for 70 s, and 100% deionized water was used as the solvent in the pre-soak method. The primary extraction method consisted of preheating for 5 min, and samples were then extracted at 70 °C under 1500 psi. Static extraction time was 5 min with a flush volume of 60% of the cell volume, purged with N_2_ for 100 sec. The solvent used for the primary extraction was a mixture of deionized water and methanol (40:60, *v/v*). Primary extraction was repeated two times. The volume of the combined extract was reduced using a rotavapor, and the concentrated aqueous extract was partitioned against ethyl acetate (EtOAc) four times. The contents of both the EtOAc phase and the water phase were examined using HPLC-DAD, HPLC-LRMS, HPLC-HRMS, and colorimetric assays including Total Phenolic Content Assay (TPC) and Total Flavonoid Content Assay (TFC). Before analysis, all phases were carefully reduced to dryness using rotavapor, and, finally, the samples were dried under N_2_ gas.

**Figure 1:**
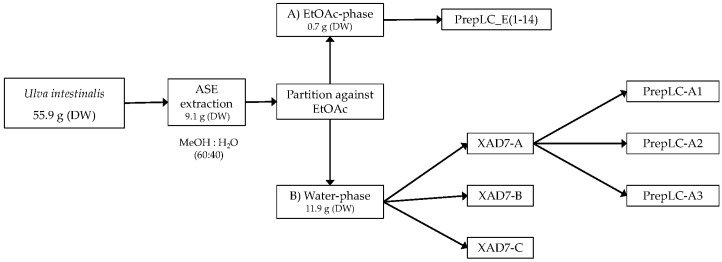
Overview of the extraction and purification steps in the *Ulva intestinalis* analysis.

The aqueous extract was applied to an Amberlite XAD-7 column and washed with distilled water. Methanol was applied for elution. The pre-eluted washing water was analyzed for polyphenols with HPLC. Collected methanolic fractions (XAD7-A, XAD7-B, XAD7-C) were reduced using a rotavapor and analyzed on analytical HPLC. The XAD-7 fraction A contained the highest number of polyphenols and was chosen to be submitted to preparative HPLC to obtain three purified fractions; prepLC-A1, -A2, and -A3 (Figure 1).

### 2.4. General Instrumentation

#### 2.4.1. Preparative HPLC

The preparative HPLC system consisted of a Gilson 321 pump (Gilson Inc., Middleton, WI, USA), an Ultimate 3000 variable wavelength detector (Dionex, Thermo Fisher Scientific, Sunnyvale, CA, USA), and a 25 × 2.12 cm (10 µm) UniverSil C18 column (Fortis Technologies Ltd., Neston, UK). Two solvents were used: (A) super distilled water (0.1% acetic acid) and (B) acetonitrile (0.1% acetic acid) with initial conditions of 90% A and 10% B followed by an isocratic elution for the first 5 minutes, and the subsequent linear gradient conditions, 5–18 min: to 16% B, 18–22 min: to 18% B, 26–31 min: to 28% B, 31–32 min: to 40% B, 32–40 min: isocratic at 40% B, 40–43 min: to 10% B. The flow rate was 15 mL/min, and the aliquots of 750 µL were injected.

#### 2.4.2. Analytical HPLC-DAD

All HPLC-DAD analyses were performed on an Agilent 1260 Infinity HPLC system (Agilent Technologies, Santa Clara, CA, USA) equipped with a 1260 diode array detector (DAD) and a 200× C analysis was performed using two solvents, (A) super distilled water (0.5% TFA) and (B) acetonitrile (0.5% TFA), in a gradient (0–10 min: 95% A + 5% B, 10–20 min: 85% A + 15% B, 20–34 min: 60% A + 40% B. 34–35 min: 95% A + 5% B). The flow rate was 1.0 mL/min, and aliquots of 20 µL were injected with an Agilent 1260 vial sampler. UV-Vis absorption spectra were recorded during the HPLC analysis over the wavelength range of 200–600 nm in steps of 2 nm.

The established HPLC method was validated for linearity, sensitivity, precision, and accuracy. Table 1 presents data for calibration curves, test ranges, limit of detection (LOD), and limit of quantification (LOQ) for gallic acid. The LOD and LOQ were calculated based on the standard deviation of *y*-intercepts of the regression line (S_y_) and the slope (S), using the equations LOD = 3.3 × S_y_/S and LOQ = 10 × S_y_/S.

#### 2.4.3. HPLC-LRMS and HPLC-HRMS

Liquid chromatography low-resolution mass spectrometry (HPLC-LRMS) (ESI+/ESI−) was performed using an Agilent Technologies 1260 Infinity Series system and an Agilent Technologies 6420A triple quadrupole mass spectrometry detector. The following conditions were applied: ionization mode: positive/negative, capillary voltage = 3000 V, gas temperature = 300 °C, gas flow rate = 3.0 L/min, acquisition range = 100–800 *m/z*. The elution profile for HPLC consisted of the following gradient: 0–3 min: 90%A + 10%B, 3–11 min: 86%A + 14%B, 11–15.5 min: 60%A + 40%B, 15.5–17 min: 90%A + 10%B, at a flowrate = 0.3 mL/min, where solvent A was super distilled water (0.5% formic acid), and solvent B was acetonitrile (0.5% formic acid). A 50 × 2.1 mm internal diameter, 1.8 µm Agilent Zorbax SB-C18 column was used for separation. Calibration curve of Apigenin ran on HPLC-LRMS and used for quantification is listed in Table 2.

Liquid chromatography high-resolution mass spectrometry (HPLC-HRMS) (ESI+/TOF) was performed using an AccuTOF JMS-T100LC (JEOL, Peabody, USA) mass spectrometer in combination with an Agilent Technologies 1200 Series HPLC system. The following instrumental settings/conditions were used: ionization mode: positive, ion source temperature = 220 °C, needle voltage = 2500 V, desolvation gas flow = 4 L/min, nebulizing gas flow = 3 L/min, orifice1 temperature = 125 °C, orifice2 voltage = 10 V, ring lens voltage = 20 V, ion guide RF voltage = 1600 V, detector voltage = 2350 V, acquisition range = 15–1000 *m/z*, spectral recording interval = 0.50 sec, wait time = 0.033 nsec, and data sampling interval = 2 nsec. The elution profile for HPLC consisted of the same gradient and column as described for HPLC-LRMS, but the flowrate was increased to 0.35 mL/min.

#### 2.4.4. NMR Spectroscopy

Quantification of the extracts of *Ulva intestinalis* was performed using ^1^H NMR analyses on a Bruker 600 MHz instrument (Bruker BioSpin, Zürich, Switzerland). All spectra were recorded in DMSO-*d*_6_ at 25 °C. The pulse sequence applied was *zg30* with the following acquisition parameters: sweep width of 19.8 ppm, 64 k data points, 16 scans, and 2 dummy scans. The relaxation delay, d1, was set to 40 sec (equal to 5 × *T*_1,max_) to ensure complete relaxation between scans. The spectra were processed using a line broadening of 0.3 Hz. The crude extract was used for *T*_1_ measurements, utilizing the *t1ir* pulse sequence with a sweep width of 19.8 ppm, 16 k data points, 8 scans, 2 dummy scans, and 9 different inversion recovery delays between 1 ms and 5 s. Measured *T*_1_ values ranged from 1.0–8.1 s.

Quantification using the ^1^H NMR spectra was performed using the ERETIC2 function in TopSpin with DMSO_2_ (10 mM) as an external reference. The DMSO_2_ signal (~3.0 ppm) was integrated and defined as the ERETIC reference (No. H = 6, Mm = 94.13 g/mol, V(sample) = 0.75 mL, C = 10 mM).

Reference compounds for validation were gallic acid (GA), *p*-coumaric acid, ferulic acid, (+)-catechin, and luteolin (10 mM, DMSO-*d*_6_). An average standard deviation of < 10% was observed. The integrations were repeated three times.

Two-dimensional heteronuclear single quantum coherence (^1^H-^13^C HSQC), heteronuclear multiple bond correlation (^1^H-^13^C HMBC), and double quantum filtered correlation (^1^H-^1^H DQF COSY) spectra were also recorded on the Bruker 600 MHz instrument.

### 2.5. Total Phenolic Content Assay

For the determination of total phenolic content, the Folin-Ciocalteu total phenolic content assay (TPC) was used. The method used was adapted from Ainsworth and Gillespie (2007) [24]. 200 µL of the sample or standard was added to the cuvettes (10 × 45 mm, 3 mL), followed by 400 µL 10% (*v/v*) Folin–Ciocalteu reagent in super distilled water. Further, 1600 µL 700 mM Na_2_CO_3_ in super distilled water was added to the cuvettes. The mixture was incubated for 30 minutes, and the absorbance was measured at 765 nm using a Shimadzu UV-1800 UV spectrophotometer and a Shimadzu CPS-100 cell positioner (Shimadzu, Kyoto, Japan). Data was expressed as gallic acid equivalents (GAE). An incubation time of 2 h was also tested.

### 2.6. Total Flavonoid Content Assay

For the determination of the total flavonoid content, 2 mL test solution (standard or sample) was added to four cuvettes (10 × 45 mm, 3 mL) and the absorbance measured at 425 nm with solvent in the reference cuvette. An aliquot of AlCl3 solution (0.5 mL, 1%, *w/v*) was added to three of the four cuvettes, and the same volume of solvent was added to the fourth (blank sample). The content of the cuvettes was stirred thoroughly, and the absorbance measured at 1 minute intervals at 425 nm for 10 minutes at 22 °C. For quantitative analysis apigenin was chosen as the reference compound (concentration range of 1–500 μg/mL). Procedure modified from Pękal and Pyrzynska (2014) [25].

## 3. Results and Discussion

### 3.1. Quantification of Polyphenols in *Ulva Intestinalis*

In this work, extraction of polyphenols was performed after optimization of extraction parameters utilizing a Dionex ASE 350 extraction instrument (see Section 2.3). Aliquots (10 mL) of the different phases, ASE (Accelerated Solvent Extractor) Crude, (A) EtOAc and (B) water (see Figure 1) were sampled and dried for weight determination and further quantification with HPLC-DAD, qNMR, TPC, and TFC. The results of the different quantification methods are shown in Table 3, Table 4 and Table 5.

### 3.2. Quantification Utilizing High-Performance Liquid Chromatography (HPLC) with Wavelength Detector (DAD)

Quantification of polyphenols in plants and foods has been a topic of discussion and research for years, and among the different methods HPLC-DAD it has been the method of choice due to the possibility of separation of compounds before individual quantification. However, with the use of retention times, absorption spectra, and molar absorptivity, the technique is often limited when it comes to simultaneous determination of polyphenols of different groups [9]. Table 6 illustrates the different area responses observed in HPLC for different standards with the same concentration, reflecting the molar absorptivity differences.

When dealing with complex polyphenolic mixtures with unknown identities, which is the case for seaweeds, one standard is often selected for quantification. Traditionally, gallic acid is chosen for total polyphenolic quantification and phloroglucinol (PGE) for total phlorotannin quantification as seen for brown algae [17,18,19,20]. In this work, gallic acid (GA) was chosen as the reference standard, since the nature of the polyphenols in the green algae *U. intestinalis* was unknown, and since we wanted to compare different quantification methods. However, there is no doubt that the estimation of the total polyphenol content will suffer from this.

The HPLC peaks with maximum intensity in the 280 nm (*R*_t_: 1–15 min) were quantified according to the 280 nm GA standard curve (Table 1), while peaks with maximum intensity in the 330 nm (*R*_t_: 15–35 min) window were quantified according to the 330 nm GA standard curve. This resulted in an HPLC-DAD quantification of 1.1% polyphenols in the algae, based on quantification on the ASE crude extract (11.3 ± 1.4 mg GAE/g DW) (Table 3). The recovery of the polyphenols after the liquid-liquid ethyl acetate partition was quantified to be 1.2% (12.1 ± 0.5 mg GAE/g DW), almost evenly distributed into the (A) EtOAc phase (0.7%) and the (B) water phase (0.6%). Thus, the total recovery for A + B was relatively close to the initial amounts found in the crude.

### 3.3. Quantitative NMR (qNMR)

**Figure 2:**
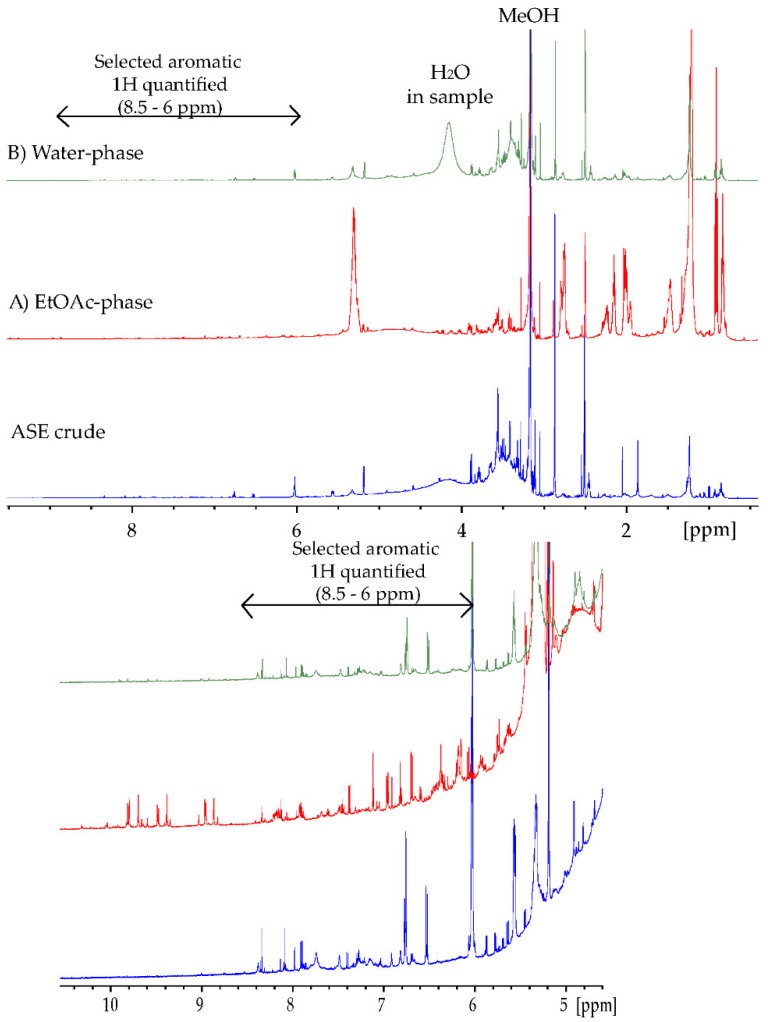
^1^H-NMR spectrum (600 MHz) for ASE crude (blue), (**A**) EtOAc phase (red), and (**B**) water phase (green) recorded in DMSO-*d*_6_ at 25 °C. 2D spectra were used to deselect peaks in the 8.5–6 ppm region belonging to the same spin system, avoiding multiple quantification.

In order to get closer to a “true” estimation of polyphenol content in seaweeds, quantifications using ^1^H NMR (qNMR) were performed (Table 4). One of the advantages of qNMR is that there is no need to consider the large variation observed regarding the molar absorptivity of different phenolic compounds (Table 6) nor the loss of sample during chromatography as with HPLC analyses. When quantifying polyphenols from NMR, one can consider two regions for quantification: the –OH spectral region, as shown by Nerantzakie et al. [23], or the aromatic 1H region [14,26]. Nerantzaki et al. presented a method for total phenolic content determination of crude plant extracts based on phenol type –OH resonances in the region between 14–8 ppm. Signals were selected after observation of elimination, or reduction, of the signal intensities after irradiation of the residual water resonance. In our marine *U. intestinalis* samples, the phenol –OH type resonances were observed at low intensities and were too broad to perform reliable integration. The broad signals may be attributed to the nature of the marine extract, containing many different types of phenol –OH resonances. Additionally, the ASE crude and the water phase contained some water, even after careful drying, which increases the phenol –OH exchange with the water peak. The 10–8.5 ppm region of the EtOAc phase (Figure 2) showed several sharp signals; however, these signals were found to not represent phenol –OH resonances due to the fact of their observed ^1^*J*_CH_ correlations in the HSQC spectrum.

For qNMR calculations, characteristic aromatic signals in the 8.5–6 ppm region of the ^1^H NMR spectra were integrated individually, and quantifications were added together to yield the total phenolic content (Section 2.4.4, Figure 2) [21,25]. Additionally, two-dimensional NMR spectra, such as COSY, HSQC and HMBC, were recorded to deselect signals belonging to the same molecule as far as possible in order to avoid multiple quantifications. The qNMR calculations were validated with quantification of standards (Section 2.4.4). Quantifications were calculated using the ERETIC2 function in TopSpin (Bruker) with DMSO_2_ as an external reference (C = 10 mM). However, to quantify the signals, a molar mass is needed. The molar mass of gallic acid was chosen in order to obtain comparable results. Quantifications were also calculated using an average molar mass of 330 g/mol based on observed masses from the MS analyses (Table 4). Additionally, an average value of aromatic protons found in each polyphenolic spin system must be chosen. This assumption will also introduce uncertainty. Nerantzakie et al. [23] made their quantification on phenol –OH and used an average of 2 OH for each spin system related to their standard, caffeic acid. In Table 4, the polyphenolic content calculation utilizing different average aromatic protons are shown, resulting in a 33% difference between the maximum (2 aromatic H) and minimum (6 aromatic H) values calculated. Based on our tentatively identified compounds in Table 7 it seemed like 4 aromatic protons (H) was a reasonable assumption. The qNMR method thus yielded a polyphenolic content of 5.3% in the crude (52.9 ± 5.2 mg 330 Mw eq./g DW). Due to the parallel numbers, using gallic acid (170 g/mol) and 2 aromatic protons yielded similar results (Table 4).

### 3.4. Colorimetric Assays: Total Phenolic Content (TPC) and Total Flavonoid Content (TFC)

The Folin–Ciocalteu assay is the most common assay used to quantify phenolic content (TPC) in both terrestrial plants and seaweeds. However, the assay is debatable due to the lack of standardization and lack of specificity in the reaction mechanism resulting in the colorimetric quantification [11,12,13,14,15,27]. This is of importance for all colorimetric assays, including the total flavonoid content (TFC) assay [25,28]. With increasing purity of the samples, direct quantitative measurements seem to be more reliable. However, the difficulty of standardizing this assay does not seem to be without importance.

The TPC assay (Table 5) resulted in a total of 0.4% in the ASE crude (5 ± 1 mg GAE/g DW), with a recovery of 0.04% in the (A) EtOAc phase (0.035 ± 0.001 mg GAE/g DW) and 0.4% in the (B) water phase (0.4 ± 0.1 mg GAE/g DW). Relatively high standard deviations were observed for the aqueous phases, potentially reflecting the lack of reliability of the method and difficulties with standardization.

The relative partition of polyphenols found between the two phases (A:B) in the TPC assay seem to follow the pattern observed from the qNMR quantification (10:90) (Table 4), rather than the partition ratio found in the HPLC-DAD analyses (50:50) (Table 3). The different ratio observed from the HPLC analyses is most likely due to the impact of molar absorptivity difference between the standard used and the compounds present.

The occurrence of flavonoids in algae is a central topic [29,30,31,32], and we chose to run a TFC assay in parallel with our attempts to identify flavonoids in our extracts (Table 8). The TFC assay gave a total of 0.03% flavonoids in the ASE Crude (0.2 ± 0.4 mg apigenin eq./g DW) and 0.13% in the (A) EtOAc phase (0.2 ± 0.4 mg apigenin eq./g DW). No flavonoids were detected in the (B) water phase with the TFC method.

### 3.5. Qualitative Analysis of Polyphenols in Ulva intestinalis

**Figure 3:**
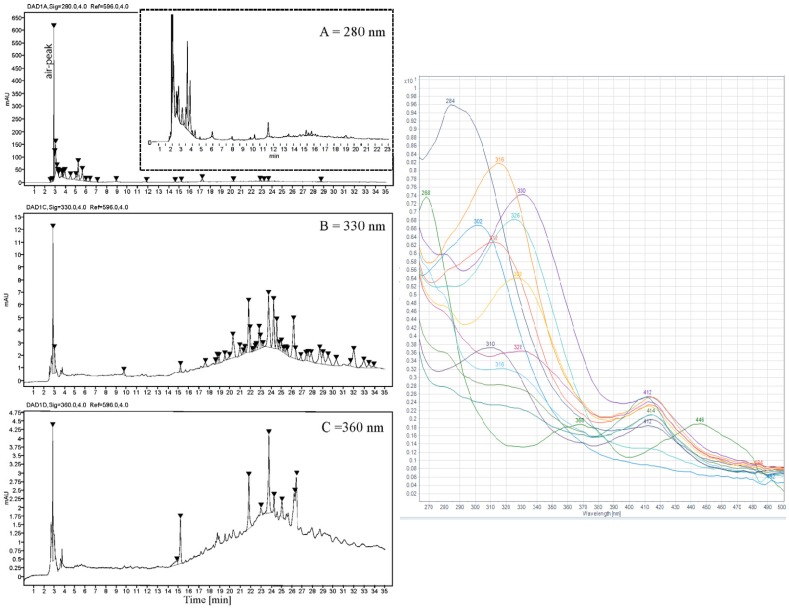
(**Left**) HPLC profile of ASE crude extract of *U. intestinalis* shown at three different wavelengths (A: 280 nm, B: 330 nm, and C: 360 nm). (**Right**) UV-Visible spectrum of selected HPLC-peaks from 15 to 35 min in the (B) 330 nm window.

**Figure 4:**
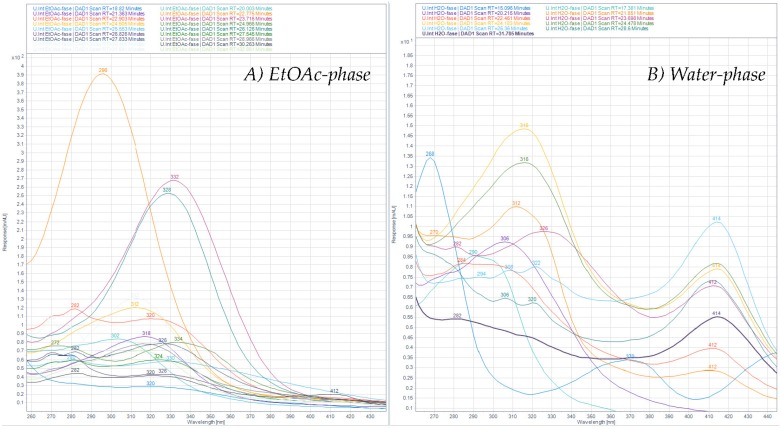
UV-Visible spectra of HPLC peaks found in the (A) EtOAc phase (**left**) and the (B) water phase (**right**) recorded at 330 nm from 15 to 35 min in the chromatograms.

After ASE extraction of the polyphenols (Figure 3; HPLC profile and selected UV-Vis spectra) and partition of the aqueous crude extract against ethyl acetate, the concentrated water phase (B) was applied to a XAD-7 column, washed with distilled water, and then eluted with methanol (Figure 1). The pre-eluted washing water was analyzed for polyphenols with HPLC-DAD. Collected methanolic fractions (XAD7 A–C) were reduced using a rotavapor and analyzed using analytical HPLC. The XAD-7 fraction A showed the highest polyphenol content and was chosen to be submitted to preparative HPLC to obtain three major fractions (prepLC A1–A3, Figure 1). The EtOAc phase was also submitted to preparative HPLC. The liquid–liquid partition with ethyl acetate gave some selectivity with respect to separation of compounds as seen in Figure 4. The compounds found in the EtOAc phase were most likely less polar and seemed to have a shorter chromophore compared to compounds observed in the water phase. The compounds in the water phase also showed an additional absorption band around 412–414 nm.

The preparative HPLC gave some separation of compounds; however, the samples were still complex. All the phases and fractions underwent extensive analyses with HPLC-DAD, HPLC-LRMS, HPLC-HRMS, and NMR. The results of the HPLC-LRMS analyses are shown in Table 7, giving an overview of the tentatively identified compounds.

Fragmentation patterns were difficult to obtain due to low concentrations. The ESI-MS spectra were recorded in both positive and negative modes. The masses of a luteolin-isomer ((M+H)^+^, calculated: 287.05556, exact: 287.05599, C_15_H_10_O_6_, Δppm 1.5) and a rhamnazin-isomer ((M+H)^+^, calculated: 331.08178, exact: 331.08178, C_17_H_14_O_7_, Δppm 1.24) were confirmed with HPLC-HRMS. The rhamnazin-isomer (*m/z* 331.08178) did not overlap with the commercial standard tricin (330 Mw) in the HPLC-LRMS SIM scan.

**Figure 5:**
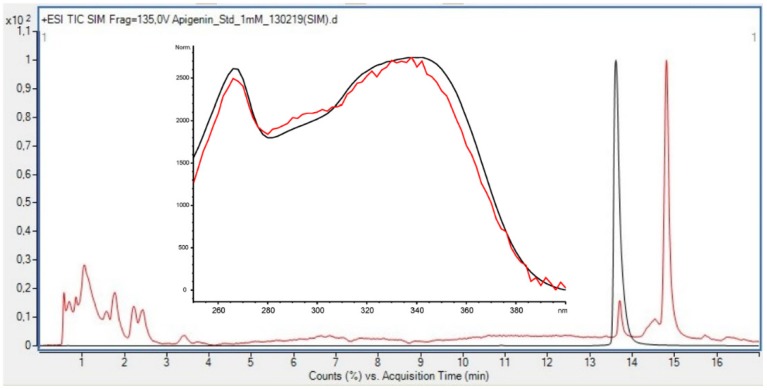
Overlaid HPLC-LRMS (+ESI) SIM Scan at *m/z* 271 of prepLC-A3 fraction (red line, C (Api, HPLC-LRMS) = 2.62 ng/g DW) and apigenin standard (C = 1.00 mM) (black line).

The most conclusive evidence of the presence of flavonoids in the green algae *U. intestinalis* was found in the late preparative fraction: prepLC-A3 (Figure 5). This fraction contained many of the peaks observed between 15 and 35 min in the HPLC profile of the crude (330 nm) (Figure 3). Several of the flavonoid masses found were tentatively identified from this fraction (Table 7) which has its origin from the water phase (B). The TFC assay did not detect any flavonoids in the water phase (Table 8) which illustrates the problem with relaying on these colorimetric assays. One flavonoid in the prepLC-A3 fraction was identified to be apigenin, using overlaid an HPLC-LRMS SIM scan at *m/z* 271 (M+H)^+^ with an apigenin standard (Figure 5). The amount of the apigenin in the algae was found to be 2.617 ng/g (DW) using an apigenin calibration curve (Table 2).

## 4. Conclusion

This case study provides an optimized extraction process for polyphenolic extraction of algae. The total polyphenolic content was quantified with qNMR (5.3%), HPLC-DAD (1.1%), and TPC (0.4%). Flavonoids and polyphenolic acids were tentatively identified in *Ulva intestinalis* samples. Apigenin was confirmed in one of the semi-purified fractions.

The same samples yielded different total phenolic contents when utilizing the different analytical methods, highlighting the difficulties related to polyphenolic quantification in extracts. All methods utilized in this study depend on assumptions and, thus, also uncertainty. This will be of special importance when analyzing complex samples at low concentrations as is the case for the polyphenolic content in marine algae. Further standardization and optimization of total phenolic quantifications of marine algae samples should be researched.

## Figures and Tables

**Table 1:** Calibration curve, limit of detection (LOD), and limit of quantification (LOQ) for gallic acid (GA) (Sigma-Aldrich) at 280 nm and 330 nm.

Standard	Calibration Curve (µg/mL)	*R* ^2^	Test Range (µg/mL)	LOD (µg/mL)	LOQ (µg/mL)
Gallic acid (280 nm)	*y* = 65.536*x* − 366.51	0.9988	10–500	14.1	42.8
Gallic acid (330 nm)	*y* = 0.2603*x* − 0.8339	0.9993	10–500	18.5	56.0

**Table 2:** Calibration curve, limit of detection (LOD), and limit of quantification (LOQ) for apigenin (Sigma-Aldrich) acquired using HPLC-LRMS.

Standard	Calibration Curve (mM)	*R* ^2^	Test Range (mM)	LOD (mM)	LOQ (mM)
Apigenin	*y* = (2.0 × 10^−6^)*x* − 2054.6	0.995	0.00156–0.0125	0.0014	0.0041

**Table 3:** Quantification of polyphenols in the crude extract and liquid–liquid extraction phases of crude with HPLC.

Sample	g DW	%PP GAE	mg (GAE)/g DW
ASE crude	9.1	1.1 ± 0.14	11.3 ± 1.4
(A) EtOAc	0.7	0.7 ± 0.2	6.7 ± 0.2
(B) Water	11.9	0.6 ± 0.1	5.5 ± 0.9
A + B	12.6	1.2 ± 0.1	12.1 ± 0.5

PP = polyphenol; (A) EtOAc = ethyl acetate phase; (B) water phase; GAE = gallic acid equivalents; DW = Dry Weight.

**Table 4:** Quantification of polyphenols in the crude extract and liquid–liquid extraction phases of crude with qNMR.

Sample	DW	GAE			330 Mw eq.			mg (GAE)/g DW	mg (330 Mw eq.) g DW
	g	%PP			%PP				
		2H	4H	6H	2H	4H	6H	4H	4H
ASE Crude	9.1	5.5 ± 0.5	2.7 ± 0.3	1.8 ± 0.2	10.6 ± 1	5.3 ± 0.5	3.5 ± 0.4	27.3 ± 2.7	52.9 ± 5.2
(A) EtOAc	0.7	0.502 ± 0.002	0.251 ± 0.001	0.167 ± 0.001	1.01 ± 0.07	0.50 ± 0.04	0.30 ± 0.03	2.51 ± 0.01	5.0 ± 0.4
(B) Water	11.9	4.9 ± 0.3	2.5 ± 0.2	1.7 ± 0.1	9.7 ± 0.7	4.8 ± 0.3	3.2 ± 0.1	24.9 ± 1.5	48.5 ± 3.3
A + B	12.6	5.5 ± 0.2	2.7 ± 0.3	1.9 ± 0.1	10.7 ± 0.4	5.3 ± 0.2	3.6 ± 0.1	27.4 ± 1.1	53.5 ± 2.1

PP = polyphenol; (A) EtOAc = ethyl acetate phase; (B) water phase; GAE = gallic acid equivalents; 330 Mw eq. = equivalents of average mass found from MS; 2H, 4H, and 6H = assumptions made related to the number of aromatic ^1^H in each polyphenolic spin system; DW = Dry Weight.

**Table 5:** Quantification of polyphenols in the crude extract and liquid–liquid extraction phases of crude with total phenolic content (TPC).

Sample	g DW	GAE %PP	mg (GAE)/g DW
ASE crude	9.1	0.4 ± 0.1	5 ± 1
(A) EtOAc	0.7	0.035 ± 0.001	0.3 ± 0.2
(B) Water	11.9	0.4 ± 0.1	3.6 ± 1.5
A + B	12.6	0.5 ± 0.1	4 ± 1

PP = polyphenol; (A) EtOAc = ethyl acetate phase; (B) water phase; GAE = gallic acid equivalents; DW = Dry Weight.

**Table 6:** Illustration of molar absorptivity differences expressed with HPLC integrated peak areas (280 nm and 330 nm) of selected standards (5 mM) used in polyphenolic quantification.

Standard	Compound Class	λ_max (nm)_	280 nm	330 nm
*p*-Coumaric acid	HCA	(230), 310	2754 ± 43	4743 ± 4
Gallic acid (GA)	HBA	272	2884 ± 2	8.7 ± 0.3
(+)-Catechin	Flavan-3-ol	279	5687 ± 6	2.1 ± 0.4
Apigenin	Flavone	(267), 340	801,120 ± 2361	131,812 ± 1525

HCA = hydroxycinnamic acid, HBA = hydroxybenzoic acid

**Table 7:** Overview of tentatively identified low-mass polyphenols/simple phenolics at different stages of purification with HPLC-LRMS.

Observed *R*_t_ (min)	(M+H)^+^	Tentative identification	LC-MS *R*_t_ Confirmed with Standard	Compound Class	Phase
1.56	171	Gallic acid	+	HBA	XAD7-A
4.74	127	Phloroglucinol *	−	benzentriol	EtOAc
6.93	291	Catechin	+	flavan-3-ol	EtOAc
8.11	181	Caffeic acid	+	HCA	EtOAc, XAD7-C
8.67	169	Vanilic acid *	−	HBA	EtOAc
9.02	165	Coumaric acid	+	HCA	EtOAc
10.10	475	Chicoric acid *	−	HCA	XAD7-B
10.27	195	Ferulic acid	+	HCA	EtOAc
10.27	183	Veratric acid *	−	HBA	EtOAc
10.51	225	Sinapic acid	+	HCA	XAD7-B
10.65	321	Luteic acid *	−	HBA	XAD7-A
12.31	475	Valoneic acid *	−	HBA	Crude, H_2_O, XAD7-A
12.50	319	Myricetin *	−	flavone	XAD7-A, prepLC-A3
12.90	287	Luteolin *^, HR^	+	flavone	EtOAc, prepLC-A3
12.98	303	Quercetin	+	flavonol	EtOAc, PrepLC-A3
13.16	273	Naringenin *	−	flavanone	PrepLC-A3
13.69	271	Apigenin (2.62 ng/g)	+	flavone	PrepLC-A3
14.43	303	Hesperetin *	−	flavanone	PrepLC-A3
14.76	289	Aromadendrin/eriodictyol *	−	flavanonol/flavanone	EtOAc
14.93	301	Diosmetin	+	flavone	XAD7-A, PrepLC-A2
14.95	303	Ellagic acid *	−	HT	XAD7-A
15.61	331	Rhamnazin *^, HR^	−	flavone	EtOAc, prepLC-A3
16.12	579	Procyanidin B1 *	−	PAC	PrepLC-A2, EtOAc
16.76	256	Chrysin *	−	flavone	Crude
16.80	317	Isorhamnetin *	−	flavonol	PreLC-A3

HCA = hydroxycinnamic acid, HBA = hydroxybenzoic acid, HT = hydrolysable tannins, PAC = proanthocyanidin. * Several possible isomers; ^HR^ HR-LC-MS mass; + = identity confirmed with standard on LR-LC-MS, - = identity not confirmed with standard on LR-LC-MS.

**Table 8:** Quantification of flavonoids in the crude extract and liquid–liquid extraction phases of crude with total flavonoid content (TFC).

Sample	g DW	mg Apigenin Equivalents	mg (Apigenin eq.)/g DW
ASE crude extract	9.1	0.03 ± 0.04 ^a^	0.3 ± 0.4 ^a^
(A) EtOAc phase	0.7	0.13 ± 0.01	1.3 ± 0.1
(B) Water phase	11.9	n.d.	n.d.
A + B	12.6	0.13 ± 0.01	1.3 ± 0.1

^a^ Three parallels measured from (0–34 mg); n.d.= not detected; PP = polyphenol; FL = flavonoid; (A) EtOAc = ethyl acetate phase; (B) water phase; TFC = total flavonoid content; DW = Dry Weight.
